# Human Temperaments. IV.—Cleverness and Capability


**Published:** 1916-06-10

**Authors:** 


### HUMAN TEMPERAMENTS.

### IV.—Cleverness and Capability.*

These two qualities are not antagonistic. They are often exhibited by the same person, and then that person is a successful person; but they are often found apart. A clever person is not necessarily incapable, nor is a capable person necessarily stupid; but still the qualities are quite different from one another; they are quite independent of one another, and may be developed to very different degrees in the same person, so that we are often surprised to find how very incapable a clever person is, and how very capable may be a person who has no claim to be considered clever.

Cleverness is much more easy to find than capability, and is much less valuable. Everyone is clever now, and cleverness is a drug in the market; but capability is far less frequent, and the demand for it never slackens. It is rather remarkable that cleverness should be so much more frequent than capability, for cleverness is an inborn quality. If a man is not born clever, no education and no training will make him clever; but anyone may train himself to become capable. Capability is, indeed, natural to some persons, and seems to be innate in them, and if not innate it may be inculcated by a proper training; but no training will make a stupid person clever.

In his mental operations the clever boy is ready, quick, nimble. His verbal memory is good, and he learns easily. At school he is high in his class. He takes a high place in examinations, and carries off many prizes. The capable boy, if he does not happen to be clever also, makes no mark at school, but he does not fail in after-life. The distinguishing character of the capable person is that he singles out the main point and sticks to it. Having once decided on a purpose he keeps that purpose steadily in view, co-ordinates all his efforts to attain it, and doggedly refuses to be diverted into side issues. Therefore he is trustworthy. What he undertakes to do he will do.

The clever man is fertile in devising new ways of meeting circumstances. He likes novelty for its own sake, and will rather try a new method that promises a great but uncertain success than the old tried method that is certainly successful but is probably tedious. A clever man may be capable also, but he is not always capable; and when he is not he is more ornamental than useful. The clever man is usually admired; the capable man is always valued. The clever incapable is a very common character, and this is much to be deplored; for, as already said, anyone may become capable who chooses to make himself so. Capability can be acquired, and it should be one of the main objects of education to see that it is acquired; but unfortunately it is but little inculcated in schools. The clever incapable, if he is a barrister, is fluent and impressive, abounds in argument, is copious in his references to cases, ready to take objections, ingenious in finding technical flaws in his adversary's case; but he does not go to the heart of the matter. His arguments, clever as they are, are not addressed to the main point; his cases are not strictly relevant; his objections, even if they are sustained, do not matter; the flaws he discovers in his adversary's case are not in the substance of the case but in the fringes. He makes a brilliant display, but he loses his cause. If he is a general, he devises a brilliant plan of campaign against his enemy, but he does not consistently adhere to it. He allows himself to be diverted from it into making discursive attacks on tempting openings—attacks that, even if they succeed, do not materially affect the campaign, and meanwhile lose time and dissipate his forces. If he is a man of science, he is fertile in hypotheses which he does not trouble to verify. As a surgeon he devises new and ingenious operations, which he executes with deftness and dexterity, for diseases that could be cured without operation. As a physician he treats in novel and striking ways symptoms rather than diseases, diseases rather than patients. In his interest in devising new ways of doing things he loses sight of the relative importance of the things to be done.

The clever shopman amuses his customers with his chatter, and surprises them with his information—the capable shopman sells his goods. The clever incapable nurse will entertain her patient with interesting conversation—when he ought to be asleep. She will arrange the flowers beautifully—and leave crumbs in the bed. She can make *pot-pourri*—but she cannot boil an egg. She knows enough about doctoring to criticise the doctor under whom she works—but there are things about nursing that she does not know. The clever person has always an excellent excuse for things going wrong or being left undone, and the clever incapable is in constant need of excuses. The capable person has no excuse ready, but then he does not need an excuse; for with him things do not go wrong, and are not left undone. If we give a job to a clever person we know that if it is done it will be done neatly and dexterously, but we have no great confidence that it will be done in time, or that it will be done at all. If we entrust it to a capable person, it may not be done as neatly or as well, but we know that it will be done, and done in time.

The clever person knows a great many things, but his knowledge is apt to be inaccurate. The capable person may not have as wide a field of knowledge, but what he does know he knows thoroughly; and though his knowledge may be confined to his work, it extends to the whole of his work. The clever incapable may have a great range of knowledge of things outside his work, but there are things about his work that he does not know. The clever man has a good verbal memory;

* The previous articles appeared on May 6, 13, and 27, 1916; and a letter on the subject on May 20.

the capable man has a good business memory. The clever man remembers what he has read; the capable man remembers what he has to do. He may forget dates, but he remembers prices: he may forget the difference between the Bill of Rights and the Declaration of Right, but he remembers the difference between a bill of exchange and a promissory note: he may forget the chemical formula of strychnine, but he remembers the difference in appearance between strychnine and Epsom salts: he may forget when the East India Company expired, but he does not forget when his licence to drive a motor-car or the lease of his house expires. He may be unable to read Greek, but he can read a map. He may be unable to write Latin, but he can write clear instructions. The clever traveller discovers a short cut to his destination, and is apt to arrive late or to lose his luggage on the way: the capable person may content himself with the old route, but he gets there in time, and carries his luggage with him. The capable traveller does not miss his train or get out at the wrong station: the clever traveller is apt to do one or the other, though he may be very entertaining to his fellow-travellers.

Clever people are apt to make mistakes and go wrong because their attention is discursive. It ranges over many subjects, and is easily diverted from the thing that matters. From this lack of concentration it results that they do not think matters out. They are deficient in foresight, and do not reckon on contingencies that are likely, but are out of the routine. Capable people concentrate their attention on the matter in hand, think it out in all its bearings, and let nothing interfere until everything likely to happen is provided for. For this reason it is not easy to take them by surprise. When there is a hitch, when things do not go as they should, the clever man has to decide on the spur of the moment, and is apt to be flustered and go wrong; but the capable man has reckoned on things turning out unexpectedly, and has made his dispositions beforehand, so that if they do, he is not taken by surprise. The clever man is apt to presume on his ability to work quickly, and so to make up for lost time, only to find that he has lost more than he can make up. The capable man does things in the order of their importance, and so is prompt, while the other is procrastinating.

An excellent example of the contrast between cleverness and capability was recently recorded in the *Times* by Dr. Broadbent. At the International Congress on Infantile Mortality held at Berlin in 1911, the German machinery for the purpose appeared to the visitors from this and other countries to be "wonderfully impressive" and "absolutely perfect." The English and American visitors were "struck dumb with admiration" and "well-nigh green from envy." The English visitors were mortified at the contrast with the methods in London or any part of the United Kingdom. But upon inquiry it was found that all this machinery, "wonderfully impressive" and "absolutely perfect" as it was, produced no result at all in diminishing the mortality among infants; whereas the methods adopted in this country, imperfect and clumsy as they appear, had actually reduced the rate of infantile mortality by 25 per cent. The Germans were clever, but the English were capable. The Germans set out to achieve a certain aim, and lost themselves in a wilderness of appliances—very clever appliances, no doubt, but appliances that did not achieve their purpose. The Germans allowed themselves to be diverted from their main purpose, of preventing infant mortality, to follow the subsidiary purpose of devising elaborate machinery; and in following this subsidiary purpose they lost sight of the main purpose. That is the mark and the characteristic of the clever incapable. He does very well indeed what is not worth doing; or if worth doing at all, is only worth doing for the sake of some ulterior purpose which he completely forgets. The English concentrated their attention on the main purpose, and achieved it. That is the difference between cleverness and capability.

